# Obesity as an Inflammatory Agent Can Cause Cellular Changes in Human Milk due to the Actions of the Adipokines Leptin and Adiponectin

**DOI:** 10.3390/cells8060519

**Published:** 2019-05-29

**Authors:** Tassiane C. Morais, Luiz C. de Abreu, Ocilma B. de Quental, Rafael S. Pessoa, Mahmi Fujimori, Blanca E. G. Daboin, Eduardo L. França, Adenilda C. Honorio-França

**Affiliations:** 1 Postgraduate Program in Public Health, School of Public Health, University of São Paulo (USP), São Paulo, SP 01246-904, Brazil; tassi.morais@usp.br; 2 Laboratory of Scientific Writing, Department of Morphology and Physiology, Centro Universitário Saúde ABC (FMABC), Santo André, SP 09060-870, Brazil; ocilmaquental2011@hotmail.com (O.B.d.Q.); faelpessoa@gmail.com (R.S.P.); mahmi_fujimori@yahoo.com.br (M.F.); bgdaboin@yahoo.com (B.E.G.D.); dr.eduardo.franca@gmail.com (E.L.F.); 3 Postgraduate Program in Public Policies and Local Development, School of Sciences of Santa Casa de Misericordia de Vitoria (EMESCAM), Vitória, ES 29045-402, Brazil; 4 Institute of Biological and Health Science, Federal University of Mato Grosso (UFMT), Barra do Garças, Mato Grosso, MT 78600-000, Brazil

**Keywords:** adiponectin, body mass index, colostrum, leptin, phagocytes, obesity, overweight, oxidative stress

## Abstract

Adiponectin and leptin play roles in the hunger response, and they can induce the inflammatory process as the initial mechanism of the innate immune response. It is possible for alterations in the levels of these adipokines to compromise the functional activity of human colostrum phagocytes. Therefore, the objective of this study is to analyze the effects of adiponectin and leptin on colostrum mononuclear (MN) cells. Colostrum was collected from 80 healthy donors, who were divided into two groups: the control group and the high body mass index (BMI) group. MN cells were used to analyze phagocytosis by flow cytometry, and reactive oxygen species (ROS), intracellular calcium, and apoptosis were assessed by fluorimetry using a microplate reader. Adipokines restored the levels of phagocytosis to the high BMI group (*p* < 0.05), with a mechanism that is action-dependent on the release of ROS and intracellular calcium. However, adiponectin and leptin simultaneously contributed to better microbicidal activity, thus reflecting an increase in the apoptosis level (*p* < 0.05) in the high BMI group. Probably, the maintenance of the balance between adiponectin and leptin levels enhances the protection and decreases the indices of neonatal infection in the breastfeeding infants of women with high BMI values. Therefore, policies that support pre-gestational weight control should be encouraged.

## 1. Introduction

Obesity is considered a complex, recurrent, and progressive chronic disease. It can be characterized as an inflammatory condition involving elevated oxidative stress, and it is associated with other diseases [1,2] due to the risk of developing comorbidities such as asthma, musculoskeletal and sleep disorders, diabetes mellitus type 2, liver and kidney dysfunction, cardiovascular diseases, infertility, and cancer [1,3,4].

It is important to highlight that women are more affected by obesity than men, which has the potential to cause an impact on the health of future generations [5,6], whether through metabolic changes transmitted to the fetus during pregnancy [6] or through the changes in an infant’s nutritional programming. Having adequate nutrition in the early stages of life is essential for the development of a child’s metabolic programming, with human milk being the most recommended food for newborns, as it provides inclusive protection against metabolic changes associated with various conditions such as obesity and diabetes. In this way, breastfeeding represents an important pathway that impacts on the reduction of excess weight in both the mother and the child [7,8,9,10,11,12], because infants who are breastfed and mothers who breastfeed have lower rates of obesity [7,10].

For the mother, breastfeeding facilitates postpartum weight loss; it is positively associated with the lean mass index and inversely associated with visceral fat thickness [9,12]. Thus, this is a way to improve a woman’s health after pregnancy, as it may help her to return to a normal metabolic profile and to lose the weight she gained during pregnancy. In infants, breastfeeding may protect against obesity through the components of human milk and behaviors related to infant feeding [8]. In any case, the programing of satiety control is one of the main breastfeeding mechanisms that helps to control obesity [12]. However, in the literature, the number of studies in this area is still scarce, so the mechanisms involved in this process have not yet been totally described.

Studies have demonstrated that metabolic changes due to maternal obesity can lead to changes in the constituents of colostrum and human milk, thereby modifying the different concentrations of hormones regulating appetite and metabolism, such as adiponectin and leptin, through breastfeeding [13,14,15,16,17]. In previously published results from this research group, it was found that the colostrum adiponectin concentration was 8.61 and 13.82 ng/mL, and the leptin concentration was 0.19 and 0.32 mg/dL in normal weight and obese groups, respectively. Furthermore, it was shown that changes in maternal serum constituents caused by maternal obesity are not necessarily reflected in colostrum constituents, especially the adiponectin levels, which were negatively correlated in colostrum and serum [15].

Adiponectin and leptin are mainly secreted by the adipose tissue and inhibit feeding by different mechanisms, acting on their respective receptors (AdipoRs and LepRs) located in neurons in the hypothalamus [18]. These adipokines levels are important indicators for the development of obesity and metabolic syndrome, since there is a reduction in the endogenous concentrations of adiponectin and an increase in leptin levels in overweight and obese individuals [19,20].

It is interesting to note that the actions of adiponectin and leptin are not restricted only to the hunger response. They also have binding sites in the cells of the immune system and activate monocytes/macrophages, triggering the inflammatory process in order to eliminate invading microorganisms. This process can also undergo alterations due to obesity and metabolic syndrome. It is emphasized that these adipokines usually act differently during the inflammatory process; adiponectin acts in a more anti-inflammatory manner, whereas leptin is more proinflammatory [3,21,22,23,24,25].

Several studies in the literature have reported that having a high maternal body mass index (BMI) is correlated with alterations in the constituents of colostrum and human milk [15,26,27,28,29]. However, the impact of maternal BMI on the functional activity of colostrum phagocytes is not entirely clear.

It is emphasized that the mother, through breastfeeding, transmits colostrum phagocytes to the infant, which are activated in the presence of microorganisms. So, the colostrum phagocytes encompass the invading particles, and during the process of phagocytosis, microbicidal activity develops via an oxidative burst, and consequently, there is an increase in the release of reactive oxygen species (ROS). Thus, colostrum phagocytes represent an additional mechanism of protection for the baby in case of neonatal infections until the infant’s immune system is developed [30,31,32].

In this study, we investigate the actions of adiponectin and leptin on human colostrum phagocytes. It is possible that changes in adiponectin and leptin in human colostrum due to maternal overweightness can lead to alterations in the responses of mononuclear phagocytes in human colostrum and compromise this fundamental mechanism of infant protection, thus increasing the risk of neonatal infection. Therefore, the aim of this study is to analyze the effects of exogenous adiponectin and leptin and their repercussions on human colostrum mononuclear phagocytes as a function of maternal body mass index (BMI).

## 2. Materials and Methods

### 2.1. Design and Sample

A cross-sectional study was carried out with the participation of 80 clinically healthy colostrum donors enrolled at the Hospital of the University of São Paulo, SP, Brazil, in 2017.The women were divided into two groups according to their pre-maternal BMI: normal BMI (18.5–24.9 kg/m^2^) and high BMI (≥30.0 kg/m^2^).

The inclusion criteria of the study were as follows: aged from 18 to 35 years; pre-gestational weight known or measured until the end of the 13th gestational week; gestational age at delivery between 37 and 41^6/7^ weeks; negative serological reactions for hepatitis, HIV, and syphilis; prenatal and non-food restrictions; and informed consent form signed. The exclusion criteria were as follows: gestational diabetes; twin pregnancy; fetal malformations; and delivery before the 36th week of gestation.

The study was approved by the Institutional Committee for Ethics in Research of the Hospital of the University of São Paulo (HU/USP) (CAAE 46643515.0.3001.0076), and all the subjects gave informed written consent before entering the experimental protocol.

### 2.2. Obtaining Colostrum and Cell Separation

About 5 mL of colostrum was collected manually from both breasts of each woman into sterile plastic tubes between 48–72 h postpartum. Colostrum was collected between feeding intervals, in the daytime period (between 10:00 and 12:00). The samples were stored at –80 °C until analysis. The experimental assays were carried out in 2017 and 2018.

The samples were thawed and then centrifuged for 10 min (160× *g*, 4 °C), which separated colostrum into three different phases: cell pellet, an intermediate aqueous phase, and an upper fat layer. The upper fat layer and the aqueous supernatant were discarded, and the cell pellet was separated using the Ficoll–Paque concentration gradient (Pharmacia, Uppsala, Sweden). The cells were resuspended in Medium 199 (Gibco, Grand Island, NE, USA) at a concentration of 1 × 10^6^ cells/mL [30,31,32] and immediately used in the assays.

### 2.3. Treatment of Mononuclear Cells with Adipokines and Zymosan

The activation of mononuclear (MN) cells was performed by incubation with Zymosan in the presence and absence of the exogenous adipokines human adiponectin (Sigma, St Louis, MO, USA) and human leptin (Thermo Fisher, Carlsbad, CA, USA), each at a concentration of 100 ng/mL. The concentrations were in accordance with data from the scientific literature [25], and preliminary pilot tests were conducted to standardize the concentrations used.

The MN cells were incubated with Zymosan (for 2 h at 37 °C under gentle shaking) and treated with 199 medium (negative control), adiponectin, leptin, and adiponectin+leptin.

The phagocytosis assays were performed with Zymosan pHrodo Green™ (Thermo Fisher, Carlsbad, CA, USA), because it emits green fluorescence in the presence of an acidic pH during the phagocytosis process. Free radical release, apoptosis, and intracellular calcium assays were performed with Zymosan (Sigma, St Louis, MO, USA) without conjugated fluorochrome to avoid interference in the fluorescence intensity of the reagents used in each assay.

### 2.4. Phagocytosis Assays

The phagocytosis assay using Zymosan pHrodo Green™ (Thermo Fisher, Carlsbad, CA, USA) did not require wash steps and quencher dyes. So, after the incubation period, 10,000 cells were analyzed by flow cytometry using FACSCalibur^™^ (BD Biosciences, San Jose, CA, USA) with excitation/emission maxima of 509/533 nm. The results were expressed by the Phagocytosis Index (%). The experiments were performed in duplicate.

### 2.5. Tests for the Analysis of Free Radicals

The cells were incubated with Zymosan in the presence of 5 μ/mL of dihydrorhodamine 123 (DHR123) (Sigma, St Louis, MO, USA). The intensity of fluorescence emitted was proportional to the amount of reactive oxygen species released [33]. The Fluoroskan Ascent FL^™^ plate reader (Thermo Scientific, Vantaa, Finland) was used, with the 485-nm excitation and 538-nm emission filters. The results were expressed as the DHR123 mean fluorescence intensity. The experiments were performed in duplicate.

### 2.6. Intracellular Calcium Assay

The cells were incubated with Zymosan in the presence of the 5-μL Fluo-3 AM solution (Sigma, St Louis, MO, USA). The cells were washed and resuspended in HBSS (Hank’s Balanced Salt Solution) containing bovine serum albumin (BSA). The fluorescence intensity was measured by a Fluoroskan Ascent FL^™^ Microplate reader (Thermo Scientific, Vantaa, Finland) using 485-nm excitation and 538-nm emission filters. The rate of intracellular Ca^2+^ release was expressed as the mean fluorescence intensity of Fluo-3. The experiments were performed in duplicate.

### 2.7. Apoptosis Assay

Cells undergoing apoptosis were detected using FITC Annexin V (BD Biosciences, Erembodegem, Belgium). The fluorescence intensity was obtained with the Fluoroskan Ascent FL^™^ Microplate reader (Thermo Scientific, Vantaa, Finland) using 485-nm excitation and 538-nm emission filters. The results were expressed as Apoptosis Index values (%). The experiments were performed in duplicate.

### 2.8. Statistical Analysis

Statistical analyses were performed with BioEstat^®^ version 5.0 software (Mamirauá Institute, Belém, Brazil). The results are presented as mean (± standard deviation). The D’Agostino normality test and variance analysis (ANOVA) were used, followed by Tukey’s test. Significant differences were considered when *p* < 0.05, and the power of the test for the sample size used was 80%.

## 3. Results

The women who had a high pre-pregnancy BMI also presented higher BMI values at the time of delivery. The other maternal/infant parameters did not differ between groups (Table 1).

**Figure 1:**
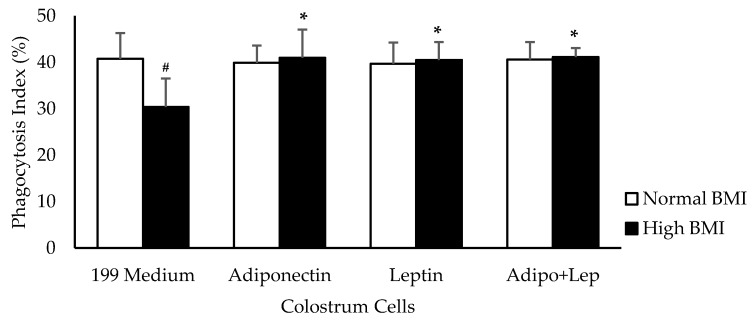
Effects of adiponectin and leptin on phagocytosis by colostrum mononuclear (MN) cells from women with normal BMI and high BMI values. Phagocytosis Index (%) values were determined by the assessment of pHRodo™ Green zymosan in MN cells from human colostrum. The results are presented as mean ± SD (n = 10 per group). They were assessed by ANOVA and Tukey’s Test. * Statistical difference between colostrum cells incubated with 119 medium and hormones within groups (*p* < 0.05). ^#^ Statistical difference among groups with the same treatment and samples (*p* < 0.05).

Pre-gestational excess weight caused a reduction in the phagocytosis index of the human colostrum mononuclear cells (*p* < 0.05). However, the addition of adiponectin and leptin (100 ng/mL) increased the percentage of phagocytosis in the group with high BMI (*p* < 0.05) (Figure 1).

**Figure 2:**
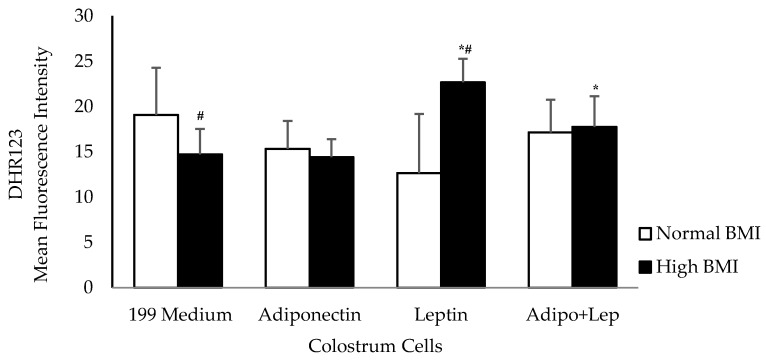
The effects of adiponectin and leptin on the oxidative burst of colostrum mononuclear cells from women with normal BMI and high BMI values incubated with Zymosan A from *Saccharomyces cerevisiae*. Reactive oxygen species were released by colostrum mononuclear phagocytes. The results are presented as mean ± SD (n = 10 per group) and assessed by ANOVA and Tukey’s Test. * Statistical difference between colostrum cells incubated with 199 medium and hormones within groups (*p* < 0.05). ^#^ Statistical difference among groups with the same treatment and sample (*p* < 0.05).

The human colostrum mononuclear phagocytes from mothers with obesity decreased the release of reactive oxygen species. When the cells were treated with adiponectin, the reactive oxygen species release was similar between groups. Higher concentrations of reactive oxygen species were observed in cells treated with leptin from the group with high BMI values (*p* < 0.05). The association with adiponectin (adipo) and leptin (lep) increased the release of reactive oxygen species when compared with untreated cells in the high BMI group, and showed similar rates of release to the cells from normal BMI individuals (Figure 2).

**Figure 3:**
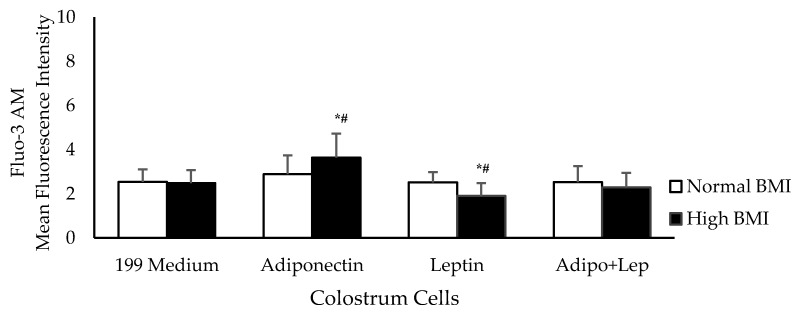
The effects of adiponectin and leptin on the release of intracellular calcium by colostrum mononuclear phagocytes from women with normal BMI and high BMI values incubated with Zymosan A from *Saccharomyces cerevisiae*. Intracellular calcium release was performed with Fluo-3AM (n = 10). The results are presented as the mean ± SD (n = 10 per group). They were assessed by ANOVA and Tukey’s Test. * Statistical difference between colostrum cells incubated with 119 medium and hormones within groups (*p* < 0.05). ^#^ Statistical difference among groups with the same treatment and sample (*p* < 0.05).

Figure 3 shows intracellular Ca^2+^ release in the presence of adiponectin and leptin. Adiponectin increased the intracellular Ca^2+^ release by colostrum phagocytes in the obesity group, whereas this was decreased by leptin. Similar rates of release were observed between groups when the colostrum cells were treated with both hormones simultaneously.

**Figure 4:**
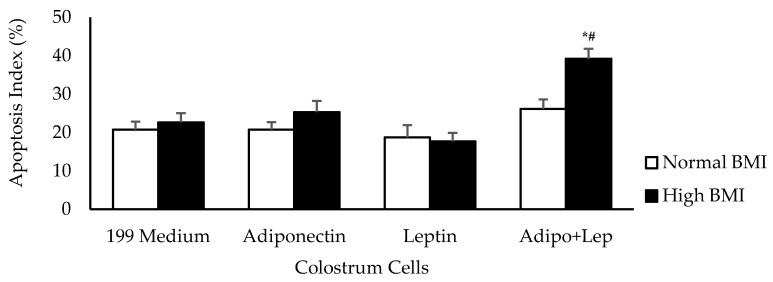
The effects of adiponectin and leptin on the apoptosis index of colostrum mononuclear phagocytes from women with normal BMI and high BMI values incubated with Zymosan A from *Saccharomyces cerevisiae*. The apoptosis assay was performed by Annexin V-fluorescein isothiocyanate (FITC) staining (n = 10). The results are presented as mean ± SD. They were assessed by ANOVA and Tukey’s test. * Statistical difference between colostrum cells incubated with 119 medium and hormones within groups (*p* < 0.05). ^#^ Statistical difference among groups with the same treatment and sample (*p* < 0.05).

Irrespective of the BMI status of the women tested, their colostrum cells showed similar apoptosis rates when treated with adiponectin or leptin compared to untreated cells. When the colostrum cells from higher BMI group were treated with both hormones, they exhibited higher apoptosis rates than in cells from normal BMI group (Figure 4).

## 4. Discussion

Obesity is characterized by an increase in adipose tissue, which is considered a large organ with plastic and endocrine properties [34]. Adipocytes secrete inflammatory cytokines and hormones called adipokines that have complex interactions with each other and are altered in overweight and obese individuals. In obese individuals, an increase in proinflammatory adipokine levels and a reduction in anti-inflammatory adipokines occurs, promoting a state of chronic inflammation [35]. The most abundant adipokines secreted by adipocytes are adiponectin and leptin [36,37].

In obese mothers, a state of chronic inflammation can trigger an exaggerated inflammatory response in the placenta, leading to the accumulation of macrophages and the production of proinflammatory mediators [38]. However, a different mechanism that leads to the production and regulation of inflammatory mediators probably operates in colostrum from obese mothers, which exhibits immunological components that protect newborns without triggering inflammatory processes [15]. This suggests that breastfeeding may represent a strategy for enhance maternal and child health, as well as controlling weight in overweight and obese individuals [7,8,12].

The findings of the present study indicate that as an inflammatory agent, obesity alters phagocytosis and reactive oxygen species release in colostrum phagocytes. In addition, colostrum cells from obese mothers showed differences in functional activity in the presence of adiponectin and leptin.

Colostral macrophages produce reactive oxygen species (ROS) and show phagocytic activity [30,31,32]. Reactive oxygen species (ROS) release is one of the last events in the course of the innate immune response and plays an essential role in the death process of invasive microorganism [39]. In this study, colostrum phagocytes from women with high BMI values were associated with lower phagocytosis index values and ROS release in the presence of Zymosan.

Several studies have reported that maternal obesity causes alterations in the components of colostrum and human milk [15,26,27,28,29]. Some authors have shown that obesity is associated with a decreased macrophage phagocytic index [40,41], whereas others suggest an increase in phagocytes and oxidative stress in these cells [42].

Interestingly, the treatment of colostrum cells with adiponectin and leptin restored the phagocytic capacity of colostrum cells from obese mothers to similar phagocytic index values to normal weight mothers. However, ROS release only showed higher levels when the cells were treated with leptin.

Leptin also plays an important role in the action of phagocytes, and can be used as a potent phagocytosis-inducing agent [43]. Immunomodulatory effects have been attributed to this hormone, which represents a link between nutritional status and neuroendocrine and immunological functions [44] because of its dual action as both a hormone and cytokine [45] that can control macrophage activation [46]. Its mechanism of action involves the activation of the JAK/STAT signaling pathways, which induce phagocytosis, oxidative burst, and the increased secretion of proinflammatory cytokines [22].

Studies have shown a significant relationship between high levels of leptin and the inflammatory state present in obesity [47,48]. Increased ROS production modifies the response of intracellular Ca^2+^ during oxidative metabolism [49]. It is important to note that although leptin is related to ROS elevation, when macrophages are in a hyperinflammatory state, they may alter the oxidative burst [21]. In this study, it is possible that the alteration in ROS by colostrum cells treated with leptin may have been associated with the reduction of intracellular calcium released by the cells from the high BMI group.

In contrast to leptin, although the presence of adiponectin in colostrum cells from obese mothers increased phagocytosis and intracellular calcium release, it did not alter the ROS release. Adiponectin, despite being known for its anti-inflammatory action, is derived from its ability to stimulate the expression of macrophage markers of the M2 phenotype. This adipokine also causes stimuli in macrophages [50,51]. Adiponectin promotes macrophage activation, inducing a proinflammatory response that resembles M1 more than M2. It induces a limited program of inflammatory activation that likely desensitizes these cells to future proinflammatory stimuli [50]. Cot/tpl2 also play an important role in the production of inflammatory mediators upon the stimulation of macrophages with adiponectin. Furthermore, activation of this axis plays an important role in the M1 proinflammatory program induced by adiponectin in macrophages [52].

Through phosphatidyl inositol 3-kinase (PI3K)/Akt, adiponectin induces the activation of the enzyme IkB kinase. This enzyme is responsible for the degradation of inhibitory protein kB and thus the consequent activation of NF-kB, which will promote the release of proinflammatory cytokines in the nucleus and trigger the inflammatory response [51].

It must be highlighted that intracellular calcium levels are fundamental for the phagocytosis process and to control the subsequent steps involved in phagosome maturation [53]. Here, colostrum cells from mothers with high BMI values treated with adiponectin exhibited higher concentrations of intracellular calcium, suggesting that it plays an important role in its mechanism of action. Intracellular calcium is responsible for the control of various cellular processes including proliferation, differentiation, and cell death [54]. Alterations in the intracellular Ca^2+^ influx by human cells may cause cell damage and have been associated with apoptosis [55].

Considering that leptin was able to activate cellular oxidative mechanisms and that adiponectin increased the calcium influx, we suggest that treatment with adiponectin plus leptin induces a more efficient phagocytic response with an association mechanism that is dependent on ROS and intracellular calcium, which culminated in increased rates of apoptosis. Possibly, this association potentiated the microbicidal activity of these phagocytes, and thus led to the consequent increase in the levels of apoptosis in the high BMI group.

According to the scientific literature, adipokines can enhance the cellular response, possibly through activation of the classical NF-kB pathway, which regulates the genes responsible for the production of most ROS in the cells [56].

From the literature, it is already known that the ratio between leptin and adiponectin is an important parameter that can be used to indicate the risk of developing obesity and even metabolic syndrome [19,20].

However, in this study, the data suggest that the actions of adiponectin and leptin on the functional activity of colostrum cells occur by different mechanisms. Adiponectin is probably associated with microbicidal activity, which is essential for the immunological response. Leptin induces high levels of ROS, which, although important for microbicidal mechanisms, may be responsible for the inflammatory processes generated by the activation of macrophages. Thus, their association is a considerable defense strategy, because in addition to inducing apoptosis, it reduced oxidative stress to levels similar to those of the non-obese group, and consequently may have controlled inflammatory processes. The maintenance of the balance of adiponectin and leptin concentrations is essential for the adequate function of mononuclear phagocytes and is extremely important for the immunologic and metabolic programming that breastfeeding can confer to the child.

This study used a cross-sectional design that brings limitations to the research. It would be interesting to develop a prospective cohort study to evaluate the effect of adipokines on human colostrum phagocytes and on the health outcomes of infants during the childhood. Such research could add important contributions that may allow the development of new strategies against the obesity epidemic, for example, through the encouragement of maternal weight control or initiatives that use colostrum and breast milk as an intervention strategy. In this sense, breastfeeding could be used at strategic hours which take into account the serum fluctuations of adipokines. Thus, we could create new alternatives with potential impacts against possible inflammatory processes caused by obesity as well as reduce the prevalence of childhood infections.

## 5. Conclusions

Pre-gestational maternal overweightness and obesity alter the functional activity of human colostrum phagocytes. In this study, the action of adiponectin on colostrum phagocytes did not alter the level of reactive oxygen species and increased the release of intracellular calcium in obese women. In contrast, leptin increased the level of reactive oxygen species and reduced the intracellular calcium concentration. The association of adiponectin with leptin was shown to be essential to restore the level of reactive oxygen species and maintain the intracellular calcium level. It also contributed to better microbicidal activity, thus reflecting an increase in the apoptosis level.

These data reinforce the importance of maternal weight control from the pre-gestational period to ensure a balance among the endogenous concentrations of adipokines and to provide to the infant, through breastfeeding, colostrum mononuclear cells that are capable of eliciting a more effective response against childhood infection. Moreover, these data support the importance of breastfeeding in the context of obesity to control the inflammatory process and reduce childhood infections.

## Figures and Tables

**Table 1:** Maternal and neonate characteristics according to maternal pre-gestational body mass index (BMI) (normal or high BMI).

Maternal and Child Characteristics	Normal BMI (n = 40) (18.5–24.9 kg/m^2^)	High BMI (n = 40) (≥30 kg/m^2^)
Age (years)	26.02 (±5.43)	25.60 (±4.88)
Diabetes or gestational diabetes (%)	00 (0.00%)	00 (0.00%)
Maternal pre-gestational BMI (kg/m^2^)	21.51 (±2.32)	31.05 (±3.77) ^#^
Delivery BMI (kg/m^2^)	25.72 (±2.32)	34.77 (±3.78) ^#^
Gestational weight gain	10.52 (±4.08)	9.11 (±3.24)
Gestational age at delivery (weeks)	38.77 (±1.10)	38.70 (±1.04)
Infant sex—female (%)	23 (57.50%)	21 (52.50%)
Birth weight (g)	3263.00 (±430.31)	3367.375 (±495.50)
Birth height (cm)	47.31 (±2.84)	47.58 (±2.69)

Maternal and neonatal data are shown as mean (±SD) or number (%). They were assessed by ANOVA and Tukey’s test. ^#^ Statistical difference among the normal and high BMI groups (*p* < 0.05).
